# A Review of Weight Control Strategies and Their Effects on the Regulation of Hormonal Balance

**DOI:** 10.1155/2011/237932

**Published:** 2011-07-28

**Authors:** Neil A. Schwarz, B. Rhett Rigby, Paul La Bounty, Brian Shelmadine, Rodney G. Bowden

**Affiliations:** Department of Health, Human Performance and Recreation, School of Education, Baylor University, One Bear Place no. 97304, Waco, TX 76798, USA

## Abstract

The estimated prevalence of obesity in the USA is 72.5 million adults with costs attributed to obesity more than 147 billion dollars per year. Though caloric restriction has been used extensively in weight control studies, short-term success has been difficult to achieve, with long-term success of weight control being even more elusive. Therefore, novel approaches are needed to control the rates of obesity that are occurring globally. The purpose of this paper is to provide a synopsis of how exercise, sleep, psychological stress, and meal frequency and composition affect levels of ghrelin, cortisol, insulin GLP-1, and leptin and weight control. We will provide information regarding how hormones respond to various lifestyle factors which may affect appetite control, hunger, satiety, and weight control.

## 1. Introduction

Obesity is a multifaceted problem with many contributing factors including but not limited to genetics, hormone levels, overconsumption of food, and sedentary lifestyle. Dietary adherence has been shown to be negatively associated with the degree of caloric restriction [1]. In light of this evidence, development of strategies to promote weight loss in the absence of purposeful caloric restriction could be advantageous in the battle against obesity. 

Since obesity is viewed as a multi-faceted problem, this paper proposes several different lifestyle factors that may be modified in order to contribute to weight reduction in the absence of caloric restriction. These lifestyle factors are macronutrient composition of meals, meal frequency, exercise, sleep, and psychological stress. In particular, this paper focuses on how the manipulation of these lifestyle factors may positively or negatively influence ghrelin, glucocorticoids (in particular, cortisol), insulin, and leptin to promote weight loss. These hormones were not chosen for review because they are the only hormones involved in weight regulation, but rather because together they are well-researched and cover a wide range of physiological functions connected with obesity and weight control. For instance, another hormone of interest in the regulation of body weight is glucagon-like peptide-1 (GLP-1). Exenatide, a GLP-1 receptor agonist, was administered to determine its effects on glycemic control and weight over an 82-week period in patients with type 2 diabetes [2]. At week 30 of the study, the amount of weight lost from baseline was 2.1 ± 0.2 kg compared to 0.3 to 0.9 kg for placebo. Reduction in body weight was progressive, resulting in an average weight loss of 4.4 ± 0.3 kg at week 82 [2]. Therefore, considering these results, the hormones in this review are not to be viewed as the only affecters of body weight regulation.

Ghrelin is a 28-amino-acid-residue peptide predominantly secreted by the stomach with substantially lesser amounts being detected in several other tissues [3]. Ghrelin appears in two major forms: acylated and nonacylated ghrelin. Acylated ghrelin is the active form of ghrelin whereas nonacylated ghrelin, which is present in greater quantities than acylated ghrelin, appears to be biologically inactive [3]. Ghrelin is a pleiotropic hormone demonstrating many different roles. Ghrelin displays strong growth hormone releasing activity through the binding to and activation of growth hormone secretagogue receptor type 1a [3]. In addition to growth hormone release, ghrelin stimulates the release of prolactin and adrenocorticotropic hormone, negatively influences the pituitary-gonadal axis, stimulates appetite, influences sleep, controls gastric motility, and modulates pancreas function [3]. Specifically, in this paper, ghrelin will be investigated in relation to lifestyle factors.

Activation of the hypothalamic-pituitary-adrenal axis results in the eventual production of the glucocorticoid known as cortisol [4]. Corticotropin-releasing hormone from the hypothalamus stimulates the release of adrenocorticotropic hormone released from the pituitary gland [4]. Adrenocorticotropic hormone is responsible for the subsequent release of cortisol from the fascicular zone of the adrenal cortex [4]. Cortisol is an adrenal steroid hormone that regulates adaptive responses to varying types of stress [5]. Specifically, in this paper, cortisol will be investigated in relation to meal frequency, exercise, sleep, and psychological stress.

Leptin is a polypeptide hormone product of the *ob* gene that is produced and secreted by adipocytes [6]. The amount of leptin produced is directly proportionate to the triglyceride content of adipocytes (i.e., the size of the fat cells) [6]. Leptin is a crucial link in the signaling process between changes in body fat and control of energy balance [6]. Specifically, in this paper, leptin will be investigated in relation to sleep.

Insulin is a peptide hormone that is released from the beta cells of the pancreas [7]. Insulin serum concentration increases with feeding and decreases with starvation [7]. Serum insulin levels are most sensitive to changes in blood glucose concentrations. Insulin binds to its receptor to initiate GLUT-4 translocation to the cell membrane in order to allow glucose to enter the cell for energy production and/or storage [7]. Specifically, in this paper, insulin will be investigated in relation to macronutrient composition of meals, meal frequency, exercise, and sleep.

## 2. Macronutrient Composition of Meals

The macronutrient composition of meals is one variable that may contribute to excessive caloric intake under ad libitum conditions resulting in unwanted weight gain and the onset of obesity. The postprandial endocrine response associated with meals of varying macronutrient proportions may give some insight as to why certain food combinations lead to greater satiety resulting in less caloric ingestion than others. Identifying and exploiting the macronutrient proportions that are associated with satiety and favorable postprandial endocrine responses may be a useful strategy for initiating weight loss through appetite regulation in the absence of purposeful calorie restriction. 

A majority of the recent studies involving the ingestion of meals of varying macronutrient proportions have focused on the resulting actions of the postprandial ghrelin and insulin response [8–13]. Both exogenous insulin administration and endogenous postprandial insulin response have resulted in suppression of ghrelin [14, 15]. In addition, faulty regulation of ghrelin suppression is associated with insulin resistance [16]. During a euglycemic-hyperinsulinemic clamp procedure, reduction of total and acylated ghrelin was greater in insulin-sensitive overweight and obese individuals versus insulin-resistant individuals of similar body type [16]. Postprandial ghrelin suppression has also been shown to be proportional to meal calorie content, and this association of meal calorie content with ghrelin suppression is reduced in obese individuals [17]. Generally, obese individuals exhibit lower fasting ghrelin levels than their normal-weight counterparts which suggests that it may not be the initial amount of plasma ghrelin that regulates appetite but the magnitude and duration of postprandial ghrelin suppression and its ensuing rise to fasting levels [13, 17]. 

The fat, protein, and carbohydrate compositions of meals have differing effects on ghrelin suppression as well as appetite regulation and satiety. In one study, high-fat meals (71% energy from fat) led to less ghrelin suppression at 30 minutes post-ingestion when compared to a high-carbohydrate (88% energy from carbohydrates) meal of equal caloric load [13]. In the same study, both lean and obese subjects reported less satiety 30 minutes after the ingestion of the high-fat meal compared with the high-carbohydrate meal. Moreover, Monteleone and colleagues [18] showed similar results in that ingestion of a high-carbohydrate meal (77% energy) more effectively suppressed ghrelin and appetite compared to ingestion of a high-fat meal (75% energy) in nonobese women. In a separate study, Foster-Schubert et al. [9] also demonstrated the inability of fats to effectively suppress postprandial ghrelin concentrations when compared to carbohydrates. In this study, however, no difference in appetite between the macronutrients was reported. Additionally, Kong et al. [11] reported that obese postmenopausal women with higher insulin concentrations had a positive correlation between saturated fat intake and ghrelin levels. Based on the results of these studies, isocalorically increasing the proportion of fat calories in relation to calories from carbohydrates and proteins is not a viable strategy to decrease appetite and ad libitum calorie ingestion. 

A study looking at the differences in ghrelin suppression and appetite sensations after either an egg (22% carbohydrate/55% fat/23% protein) or bagel (72% carbohydrate/12% fat/16% protein) breakfast found an inability of either meal to suppress ghrelin [19]. In fact, ghrelin levels were increased after both meals although the rise was significantly less after the egg breakfast. Despite the unexpected increase in ghrelin, the egg breakfast was associated with significantly less “hunger” and greater feelings of satisfaction than the bagel breakfast at three hours after ingestion. Also, consumption of the egg breakfast resulted in a lower overall energy intake compared to consumption of the bagel breakfast during the subsequent 24-hour period. The researchers credited the feelings of satiety associated with consumption of the egg breakfast to the suppressed ghrelin response and tempered insulin/glucose fluctuation [19]. Examination of other research studies suggests that the increased satiety and subsequent decrease in energy intake might be at least partly explained by the higher protein content (23% of calories for egg breakfast versus 16% of calories for bagel breakfast) of the egg breakfast [8, 10, 20]. Specifically, evidence from Leidy et al. [21] supports the idea that a high-protein intake (25% of meal calories from protein) at breakfast may be important for sustained fullness during periods of energy restriction. Conversely, when a high-protein breakfast (58.1% protein, 14.1% carbohydrate) was compared to a high-carbohydrate breakfast (19.3% protein, 47.3% carbohydrate), no difference in subsequent appetite or ad libitum caloric intake during lunch was observed [22]. However, a larger suppression of postprandial ghrelin was observed for the high-protein breakfast [22]. Numerous studies provide evidence that supports the use of a high-protein diet to induce appetite suppression and decreased ad libitum energy intake. Weigle et al. [20] reported that an increase of protein from 15% to 30% of dietary intake resulted in a decrease of ad libitum caloric intake and weight loss. These effects were evident in the absence of ghrelin suppression. Beasley et al. [8] also reported that a meal with 25% protein suppressed appetite (compared to a 15% protein, high-fat group and a 15% protein, high-carbohydrate group). Similar to the results presented by Weigle et al. [20], reduction of appetite could not be explained by a suppression of postprandial ghrelin [8]. The likely mechanism of action of high-protein diets on appetite suppression and decreased caloric intake may be outside of the control of ghrelin. This is evidenced by the mixed results of studies reporting protein's suppression of postprandial ghrelin with some researchers reporting large differences in postprandial suppression of ghrelin [9] and others reporting no differences in postprandial suppression of ghrelin [8, 10, 20] when high-protein meals are compared to meals of lower-protein composition.

In addition to a relatively high protein intake (~30% of total calorie intake), moderate carbohydrate intake may also be important in regulating sensations of hunger and calorie ingestion. As mentioned earlier, endogenous insulin secretion may result in the suppression of ghrelin [15]. In a recent study of healthy Pima Indians, higher plasma insulin responses were associated with a decrease in subsequent carbohydrate consumption and less weight gain [23]. These results further point to insulin as a regulator of calorie consumption. While, in one study [24], a short-term isocaloric high-carbohydrate diet was not associated with a lower overall energy intake when compared to a high-fat diet, another study [25] demonstrated that a high-protein meal (35% of total calories) with moderate carbohydrate intake (45% of total calories) was able to persistently suppress ghrelin at levels significantly lower than baseline when compared to either a mixed-diet (50% carbohydrate, 20% protein) or high-fat (45% carbohydrate, 45% fat) meal. It is unclear whether the glycemic index or glycemic load plays a direct role in the regulation of appetite and subsequent energy intake through tempered insulin responses as results from past studies have been conflicting [26, 27]. Furthermore, insoluble fiber at high doses (33–41 grams) may help to regulate appetite as well as energy intake [28, 29]. 

In conclusion, evidence tends to suggest that for the regulation of ad libitum caloric ingestion, a high-protein diet (~30% of total calories) may be beneficial whereas high-fat diets should most likely be avoided. It would appear to be most beneficial if the additional protein is ingested from solid food sources as food form also appears to play a role in feelings of satiety [30, 31]. Future research should investigate the effects of large doses of insoluble fiber (33 to 41 grams) in addition to a high-protein diet to determine if this combination could further attenuate appetite and energy intake.

## 3. Meal Frequency

Another factor associated with weight control that may not necessarily have a concomitant decrease in caloric intake is meal frequency. Caloric intake can be affected by caloric density in food, total energy consumption as well as meal frequency, but Solomon et al. [32] suggests that feeding frequency has received the least amount of empirical research. Additionally Stote et al. [33] most recently reported that nutrient-dense and low-calorie diets have received significant attention with regards to weight control and health outcomes, but the influence of meal frequency is yet to be firmly established. Though the general public would suggest that three meals a day are important [33] there have been few well-controlled studies that have compared meal frequency, with the same caloric consumption, and its effect on health outcomes and weight control. To date evidence suggests that less frequent meal consumption with a large bolus of calories at each meal can lead to increases in adipose tissue [34]. Conversely consuming the same amount of calories with more frequent and smaller meals does not seem to impact the deposition of fat [34]. Additionally Solomon et al. [32] report an increase in snacking has positive associations with BMI, but also with caloric intake, suggesting the need for studies involving meal frequency and weight control. 

There have been some published findings regarding meal frequency and its effects on preprandial and postprandial satiety and gastric sensations of “fullness” or “emptiness” with some studies suggesting hormones may play a direct or indirect role [35]. These hormonal effects may primarily be related to ghrelin and insulin and possibly cortisol [33, 35]. 

Much of the research regarding weight control and hormones has focused principally on ghrelin, a hormone produced primarily in the gastrointestinal tract with larger amounts in the stomach [35]. Recent findings have suggested that ghrelin may play a role in the control of food intake and meal frequency as well as energy intake [36]. Specifically ghrelin in the plasma increases preprandially in fasting conditions and before meal initiation and will rapidly decrease nadir postprandially [37] and therefore is reported to play a role in appetite regulation, meal frequency, and hunger [36]. Solomon et al. [32] have suggested that a larger preprandial surge in ghrelin may occur with larger and less frequent meals that could cause more meal initiation and more between-meal snacks. Other study authors have reported that ghrelin may play an important role in metabolic balance by decreasing fat utilization and increasing appetite and meal frequency [38]. The same authors report that this metabolic role has occurred in both healthy populations and in cancer patients suffering anorexia. 

Another hormone thought to regulate appetite is insulin. Postprandial insulin levels have been more controlled with more frequent meals, with meal skipping (primarily skipping breakfast) associated with higher levels of insulin [39], but most of the research regarding insulin and ghrelin has focused on the interaction of the two hormones and how each may affect each other in appetite control and meal initiation and frequency. 

Postprandial suppression of ghrelin has been demonstrated to be partially dependent on the release of insulin [32, 40] and can be subject to insulin sensitivity. Previous reports [41] have suggested that the administration of ghrelin in apparently healthy participants has an inverse relationship with insulin levels and causes an increase in plasma glucose concentrations. Other study findings [32] also suggest that insulin and ghrelin have an inverse relationship in fasting conditions as well as with low-frequency meal ingestion, causing poor control of insulin and decrease in glucose consumption [41], yet when meals are consumed more frequently with the same caloric consumption, the insulin-ghrelin relationship is less apparent. The lack of a relationship with high frequency meals may be due to less insulin-related fluctuations in ghrelin and therefore may cause there to be less of a preprandial increase in ghrelin and consequently less gastric sensations or “hunger” [32, 42, 43]. Conversely, an increase in feeding frequency that is associated with an increase in caloric consumption may cause the insulin to exert less control on ghrelin causing an increase in preprandial ghrelin [42]. This can be especially true in Type II diabetics [32]. Postprandial ghrelin suppression and downregulation in insulin-resistant disease states such as diabetes and obesity and in syndromes such as metabolic syndrome is lesser than in apparently healthy populations [32, 34, 44].

A third hormone that may have an effect on appetite control, meal frequency, and regulation is cortisol. One study [36] reported that cortisol levels correlated negatively with ghrelin suggesting that cortisol may peak after ingestion of a meal and return to nadir some time after meal completion. Hypercortisolism in another study [45] suppressed ghrelin levels. 

Therefore a novel approach to weight control in the absence of direct caloric restriction could be in the control of ghrelin levels. One study [46] concluded that a ghrelin mimetic increased appetite transiently and that the infusion of ghrelin countered the effect of anorexia in elderly persons. Similarly, other studies suggested a ghrelin mimetic helped to increase appetite and caloric consumption in both end-stage renal disease [47] and cancer patients [38]. Since it has been established that both insulin [36, 46, 48] and cortisol [33, 36] have negative and regulatory effects on the levels of ghrelin, a novel approach for weight control and weight loss would be to attempt to control ghrelin levels by attempting to moderate the preprandial increase. Such control could cause a concomitant decrease in appetite with the possible implication of caloric restriction due to lower levels of hunger. Control of ghrelin, behaviorally, could occur through the increase of meal frequency and smaller meals. The same amount of calories in smaller more frequent meals can help to regulate glucose and therefore insulin and cortisol and indirectly affect the levels of ghrelin in plasma and ghrelin secretion. Soule et al. [48] demonstrated that an injection of ghrelin was associated with an increase in calorie intake in one meal by 30%. Conversely, any attempts to control ghrelin endogenously may have the opposite effect and help reduce the caloric consumption in each meal. Combining both an increased meal frequency with endogenous control of ghrelin could help in the decrease of caloric consumption. More research is needed to confirm these findings with longitudinal outcomes measured.

## 4. Exercise

The response of total plasma ghrelin to bouts of exercise has been investigated in humans [49–52], horses [53, 54], and rats [55, 56]. In humans, studies reporting the observed levels of ghrelin following acute bouts of exercise are conflicting. More specifically, ghrelin concentrations remained constant during a single bout of treadmill running for one hour at 73.5%  VO_2max_ in healthy, physically fit individuals [57] and during rigorous running at increasing intensities in endurance athletes [58] and healthy volunteers [59]. Moderate-intensity resistance exercises in which both eccentric and concentric contractions were performed [60] and acute incremental exercise in healthy men [61] have shown decreases in ghrelin concentrations [62]. 

While it is inconclusive how ghrelin concentration alone changes in response to an acute bout of exercise, ghrelin can potentially stimulate the release of growth hormone (GH) during exercise [50]. Since the process of releasing GH occurs through the activation of the GH secretagogue receptor [63], and GH increases during acute bouts of exercise, the relationship between exercise, plasma ghrelin levels, and GH has been investigated [50]. In most studies, GH levels increased while plasma ghrelin levels remained unchanged regardless of the duration or intensities of the exercise protocols [49, 58–60]. It is suggested that under these conditions, peripheral circulating ghrelin does not mediate pituitary GH secretion [50]. However, there are studies that contradict the results of the aforementioned studies. For example, a study by Borer et al. [64] reported an increase in the plasma concentration of both ghrelin and GH following exercise energy expenditure in overweight, postmenopausal women [64]. Christ et al. [65] also observed increases in ghrelin and GH concentrations in athletes following a three-hour bout of aerobic exercise at an intensity of 50%  VO_2max_ [65]. 

Ghrelin concentrations have also been investigated concurrently with exercise, hunger, and food intake responses. Recently, Erdmann et al. [50] examined the effect of a continuous bout of exercise (for up to two hours), performed at low to moderate intensity, on food intake. One group of subjects exercised with a bicycle and performed one control protocol and two uninterrupted exercise protocols: cycling for 30 min at a low intensity (50 W) and cycling for 30 min at a moderate intensity (100 W). Though there was an initial increase in ghrelin concentration observed during the first protocol, the concentration remained unchanged during the second protocol. Hunger and food intake responses were not significantly different from the controls. Another group of subjects exercised with a bicycle and performed one control protocol and three uninterrupted exercise protocols: cycling for 30, 60, and 120 min, all at low intensity (50 W). Ghrelin levels increased by 50–70 pg/mL above baseline for the respective bouts of exercise. Moreover, food intake response after 120 min of bicycle exercise was significantly greater than the controls and compared to the first two exercise protocols [50]. This study suggests that duration has more of an impact than intensity with respect to an increased ghrelin response after a continuous bout of exercise. 

Studies that utilize long-term exercise protocols have shown ghrelin concentrations to increase in response to the subsequent exercise-induced decrease in body weight that acts via a negative feedback loop that controls body weight [51, 52, 66]. Circulating ghrelin levels have demonstrated an increase over time in healthy women who lost weight during a three month, energy-deficit-imposing diet and exercise regimen [52]. Also, it has been reported that total plasma ghrelin increases by 18% in sedentary, overweight, postmenopausal women who lost more than 3 kg of body weight after a 12-month aerobic exercise protocol [51]. However, Morpurgo et al. [67] conducted a three-week exercise training program that reduced body weight in obese patients and found that fasting and nonfasting ghrelin levels were unchanged at the conclusion of the program compared to levels observed before the program began [67]. 

Much like the ghrelin response, the insulin response is also sensitive to acute bouts of both endurance and resistance exercise. Endurance exercise promotes oxidation in the skeletal muscle, which is a major component in the mediation of insulin action [68–71]. Although an acute bout of endurance exercise enhances insulin sensitivity [72–74], it has been reported that an acute bout of sprint interval exercise has no effect on insulin sensitivity in healthy subjects [75]. 

Since insulin controls the metabolism and uptake of glucose in muscle cells and mediates blood glucose levels, serum insulin concentrations correlate with fluctuations in blood glucose [76]. This response is increased when proteins and carbohydrates are consumed at any stage of a workout, including pre-, mid-, and postworkout [77–80]. However, if a protein/carbohydrate supplement is not ingested at any stage during resistance exercise, it has been demonstrated that serum insulin levels slightly decrease [81]. For example, Raastad et al. [81] observed a 3% decrease in insulin concentrations compared to baseline values 30 min after a moderate-intensity resistance exercise protocol in male athletes. The same study also observed a 4% decrease in insulin concentrations compared to baseline values 30 min after a high-intensity resistance exercise protocol in the same subjects, revealing that there was no significant difference in the levels of insulin when the two protocols were compared [81]. 

Resistance exercise protocols that utilize hypertrophy lifting strategies also show that the acute insulin response remains unchanged [79, 82–84]. However, if a protein/carbohydrate supplement is ingested prior to the workout, there is a significant increase (up to a 5-fold increase, on average) in the insulin response following exercise [85]. Williams et al. [84] observed that insulin levels only rose significantly in resistance-trained men after ingestion of a carbohydrate-protein supplement compared to when the same subjects ingested a placebo following bouts of resistance exercise using a load equal to each subject's 10-RM [84]. Dietary intake that includes consumption of any combination of amino acids and carbohydrates during a stage of resistance exercise maximizes insulin's influence on tissue anabolism and protein synthesis by decreasing protein catabolism [77, 80] and utilizing enhanced blood flow in the skeletal muscles [76]. 

Hypertrophy schemes and other resistance exercise protocols are known to produce acute increases in other hormones, without supplementation, including the catabolic hormone cortisol [85]. The antagonistic effects of catabolic hormones and anabolic hormones, such as testosterone, promote muscle growth and protein metabolism during rest in between workouts [86–89]. These processes are accomplished through the inhibitory actions of cortisol in the synthesis of contractile muscle proteins [90], as opposed to testosterone, which has the opposite effect [91, 92], resulting in increased muscle mass [93, 94]. Furthermore, cortisol promotes triglycerides to be hydrolyzed into free fatty acids and glycerol [95–97], thereby allowing for increases in exercise performance and recovery [98]. Circulating cortisol can also be a source for energy production, as high levels of the hormone can initiate gluconeogenesis in the liver [97]. Increases in cortisol concentration may also lead to an increase in appetite and energy intake. This was demonstrated by Tataranni et al. [99] who showed that a therapeutic dose of glucocorticoids increased appetite and energy intake of healthy male subjects. Conversely, low cortisol concentration results in hypophagia and possibly decreased energy intake [100].

An acute bout of resistance exercise has been shown to cause significant increases in cortisol in men although the changes observed in the hormone levels are influenced by intensity, duration, muscle mass and loading schemes, and the degree to which the subject has been trained for this type of exercise [94, 101]. Increases in circulating cortisol concentrations are typically proportional to the intensity of the exercise performed, but reach a maximum threshold value that is dependent upon the duration of the exercise protocol [96, 98]. 

Increases in circulating cortisol levels have also been observed in women after an acute bout of resistance exercise [94] and have similar responses to men performing the same protocol [102]. However, one study by Häkkinen and Pakarinen [103] showed no statistical change in cortisol levels among women in three different age groups (30 years, 50 years, and 70 years) who performed the same heavy resistance exercise protocol while a significant increase in cortisol was observed in the middle-aged men compared to the younger and older men, who performed the same heavy resistance exercise protocol and were classified with respect to age, in the same manner as the women who participated in this study [103]. 

With regards to an acute bout of endurance training, cortisol normally increases with respect to exercise intensity [104–106], but the correlation is not necessarily linear [107]. Circulating cortisol concentrations have been demonstrated to increase in trained men after repeated 100m sprints [108] and increase in type 1 diabetics during and after sprinting exercises [109]. Moreover, cortisol levels have been reported to increase in short (7 min) and longer (40 min) bouts of rowing, an exercise that combines both aerobic and anaerobic actions [110]. Although no change or even a reduced change in cortisol concentrations at low exercise intensities has been reported [98], this observation has been refuted [107]. Similarly, the effects of acute bouts of high-intensity endurance exercise in athletes are conflicting. One study reported no change in cortisol concentration in endurance-trained athletes following 10 min of moderate- to high-intensity running [111], but another study reported an increase in cortisol concentration in male athletes following a ramp incremental cycle ergometry exercise to exhaustion [112]. 

The cortisol response to exercise is also influenced by subject training status. In untrained men, postexercise cortisol concentrations, measured after one, six and eight weeks from starting a heavy resistance exercise protocol, increased compared to preexercise values while resting cortisol concentrations decreased at eight weeks from starting the program [102]. In moderately trained men, cycling at moderate to high intensities provokes increases in circulating cortisol concentrations, while cycling at low intensities reduces circulating levels of the hormone [98]. When comparing long-term trained to untrained middle-aged men, cortisol levels increased only in the untrained group after performing multiple sets of a superset strength training protocol at 75% of 1 repetition maximum, which suggests that long-term, strength-trained men need a higher-volume exercise protocol in order to counteract the lower responsiveness in cortisol values observed in this study [113]. No changes have been observed in cortisol concentrations among untrained females who trained for eight weeks and were divided into four groups: control, endurance exercise (up to 80% MHR by the 8th week), resistance exercise (up to 80% 1RM in three sets and six repetitions), and concurrent exercise [114].

Cortisol concentrations also differ among trained subjects who have different training histories. For example, cortisol levels are less pronounced in endurance-trained subjects compared to resistance-trained subjects who performed the same exercise program, which included both endurance and resistance exercises [115]. Other factors that can influence the observed elevations in circulating cortisol concentrations during resistance exercise include plasma volume reductions (when corrected still result in elevated cortisol levels) [116] and anabolic steroid use [117]. 

Exercise can help to control weight through an increase in the number of calories expended. The amount of calories expended during exercise has an equivalent effect on body composition as the same amount of calories being restricted from the diet with the added benefit of cardiovascular and muscular conditioning [118]. In fact, substantially increasing the amount of calories expended by exercise during weight loss may lead to better long-term weight control and markers of health [119]. However, one negative result of such high exercise volume may be the accompanying rise in cortisol. Such an increase in cortisol could possibly be blunted through nutritional supplementation of fish oil and phosphatidylserine. Noreen et al. [120] were able to demonstrate that supplementation with 4 grams of fish oil daily (1,600 mg/d eicosapentaenoic acid and 800 mg/d docosahexaenoic acid) led to increased fat free mass and decreased fat mass in men and women after 6 weeks compared with 4 grams daily of safflower oil. The fish oil supplementation did not result in a statistically significant decrease in salivary cortisol compared with the placebo group, but there was an existing trend. The confidence intervals for pre- and postmeasurements of salivary cortisol after fish oil supplementation (−0.127 *μ*g/dL, −0.002 *μ*g/dL) support the trend that fish oil supplementation may lower cortisol concentration [120]. The proposed mechanism of lowering cortisol is through a decrease of IL-6 which has been shown to be a stimulator of the hypothalamic-pituitary-adrenocortical (HPA) axis independent of corticotropin-releasing hormone (CRH) activity [121]. In another study, short-term phosphatidylserine supplementation of 600 mg daily was able to blunt increases in cortisol induced by training in athletes [122]. Ten male subjects were administered phosphatidylserine or placebo for 10 days and then subjected to a moderate-intensity treadmill exercise protocol. Before the postsupplementation testing session, basal cortisol levels were lower than presupplementation levels for the phosphatidylserine group compared with placebo. Exercise-induced cortisol response was also reduced by 39% in the supplement group compared to placebo. Testosterone levels in the phosphatidylserine group also increased compared to the placebo group, but these results were not statistically significant. The resulting testosterone to cortisol ratio for the phosphatidylserine group was improved which may lead to further positive physiological consequences [122]. The findings of this study suggest that phosphatidylserine is effective in attenuating the cortisol response to exercise and may potentially prevent the physical deterioration that can result from increased training volume [122]. 

In conclusion, exercise, especially at higher volumes, is an important part of any weight loss regimen. Exercise may exert its weight control benefits through its positive effects on insulin and ghrelin. Exercise can also help one adapt to the physiological effects of cortisol, but, exercising at very high volumes in an attempt to maximize energy expenditure may lead to an excessive cortisol response. Therefore, blunting the cortisol response associated with very high volumes of exercise through the use of fish oil and phosphatidylserine supplementation may be a viable option.

## 5. Sleep

An often-overlooked aspect of weight control is that of sleep duration. An inverse relationship between sleep duration and body mass has been reported in both adult [123] and adolescent [124] populations as well as in different cultures [125–129]. Since metabolism, endocrinology, and circadian rhythms are tightly linked, this finding should be intuitive. 

While there are several hormones that affect weight control, leptin, ghrelin, cortisol, and insulin are a few affected by sleep. Given the roles each hormone plays in metabolism or appetite control, learning how to manipulate the hormonal response behaviorally could be of benefit in managing weight control with or without caloric restriction. The effects of these hormones are interconnected, but each hormone will be briefly papered independently to elucidate the effects sleep has on each.

Well-controlled studies have demonstrated the effect of sleep duration on leptin. Under normal, healthy circumstances, leptin levels peak between midnight and early morning with a nadir between midday to midafternoon [130]. Thus the peak occurs during the dark phase of the 24-hour cycle and the nadir during the bright phase. Sinha et al. [130] suggested that this nocturnal elevation in leptin was to suppress appetite during normal sleeping hours. Interestingly, changes in the light/dark cycle and meal timing have been shown to alter plasma leptin levels [131]. When subjects underwent a 12-hour time zone shift, analogous to inverting the light/dark cycle, peak and nadir leptin levels were shifted by about 12 hours [131]. Furthermore, leptin peaks and nadirs were shifted, yet again, when meal consumption was altered by 6.5 hours [131]. Additionally, chronic sleep deprivation has been shown to reduce the amplitude of diurnal variation with leptin [132]. Similar reductions occur in leptin levels and amplitude of diurnal variation when sleep duration is reduced from 8 hours to 4 hours [133]. This phenomenon has also been reported in a large population-based study where short sleep duration was associated with low leptin levels [134]. The same population-based study reported shortened sleep duration to be associated with high ghrelin levels and increased BMI [134]. 

While ghrelin levels increase in the fasting state and are known to decrease shortly after food consumption [135], levels are also known to peak during the night and decrease before waking hours [135–137]. Relative to sleep, ghrelin has been shown to promote slow-wave sleep (SWS), which is important for recovery of metabolic function [138]. In sleep-deprived states, the nocturnal rise in ghrelin was blunted [136]. Furthermore, Dzaja et al. [136] reported that although the peak response was blunted in the absence of sleep, ghrelin levels steadily increased until early morning hours and remained elevated until after breakfast hours. Given ghrelin's pleiotropic nature (i.e., its effects on appetite, stimulation of growth hormone, and promotion of SWS in normal situations), the loss of sleep seems to have multiple effects on ghrelin levels. If sleep duration is shortened and peak levels of ghrelin are blunted but those levels do not fall to nadir until after normal breakfast hours, the result could be an increase in appetite leading to increased caloric consumption. Additionally, it has also been demonstrated that growth hormone secretion would be blunted [136]. It is believed that ghrelin may act in a synergistic fashion with growth-hormone-releasing hormone [139] as well as act as an interface between the hypothalo-pituitary-adrenocortical system and the hypothalamo-pituitary-somatotrophic system [138]. In accordance with this role as an interface between these two systems, ghrelin not only increased the duration of SWS sleep, but it also increased cortisol levels [138]. 

As has been reported for leptin and ghrelin, a relationship between sleep and cortisol levels exists [133, 140, 141]. In normal, healthy conditions, cortisol levels decline prior to sleep onset and rise again during late night hours reaching a peak during early morning [142, 143]. During acute sleep restriction cortisol levels were reduced in early morning hours [141, 144], but increased during evening hours when leptin levels were blunted [133]. This relationship points to the interaction between cortisol and leptin and a possible role for sleep on weight control via hormonal regulation. While cortisol increases leptin production in a dose-dependent manner [145], the regulation of leptin by glucocorticoids is not absolute [146]. Conversely, leptin has a suppressive effect on the HPA axis [147]. Thus, a feedback loop exists between the two. Additionally, cortisol increases food consumption [99]. The cortisol and leptin response to reduced sleep duration would indicate that in a sleep deprived state, as leptin levels fall, which has a decreased effect on appetite suppression, concomitant increases in cortisol may cause an increase in food consumption [133]. The cortisol response to acute sleep restriction appears to be similar in a chronic situation. In a large study using self-reported sleep duration, sleep restriction and high sleep disturbance resulted in elevated cortisol levels during evening hours [140] supporting the results observed in the acute studies.

Finally, sleep loss has been proposed as a risk factor for insulin resistance and type 2 diabetes [148]. After six days of sleep restricted to 4 hours, peak glucose responses after breakfast were elevated indicating decreased glucose tolerance [149]. The decrease in glucose tolerance was also reported in a follow-up study where subjects underwent two days of restricted sleep where glucose levels were elevated while insulin levels were lower [148, 150]. Recent epidemiological studies support the acute findings as decreased sleep duration has been reported as an increased risk factor for impaired fasting glucose [151] and impaired glucose tolerance [152].

Based on these aforementioned studies, it would be apparent that increasing sleep duration could be a valid approach to weight control with or without caloric restriction as well as metabolic complications associated with obesity. However, research on the effects of increased sleep on weight management is limited. Recently a feasibility study on this very topic was reported and is underway, but final data has not been reported [153]. Thus, more studies are needed to ascertain whether increasing sleep duration is an effective tool in controlling obesity.

## 6. Psychological Stress

Another one of the factors that may contribute to weight gain and obesity is psychological stress [154]. Increased levels of perceived stress are thought to influence eating behavior [155]. In fact, in a recent paper it was reported that during stressful conditions approximately 40% of people eat more food, 40% consume less food, and 20% do not alter their eating patterns [155]. Tataranni et al. [99] demonstrated that individuals who were given an exogenous glucocorticoid ate significantly more food throughout the day as compared to participants who were only given a placebo. Moreover, during periods of increased stress, certain individuals may choose foods that are higher in fat and sugar (i.e., comfort foods). The ingestion of these comfort foods is thought to improve mood and decrease stress via opioidergic and dopaminergic neurotransmission [156]. Thus, consuming these foods can be construed as a type of coping mechanism to mitigate stress in certain populations. However, if these energy-dense food choices are consumed chronically in response to stress, it could theoretically lead to increased food consumption and ultimately weight gain [156]. The influence of psychological stress on various hormone levels and their effect on hunger and weight management are areas of interest in obesity research. In particular, cortisol and ghrelin are two hormones that are possibly thought to influence eating following a stressful event [157]. 

Chronic-stress-induced secretion of cortisol and/or increased HPA axis activation may specifically contribute to central/abdominal obesity [158, 159]. Cortisol appears to affect visceral fat to a greater degree than subcutaneous fat due to abdominal adipose tissue having increased cell density and more glucocorticoid receptors [158]. Central fat, also known as visceral obesity, is particularly undesirable because it is associated with hypertension, cardiovascular disease, and diabetes [158]. Interestingly, the mental stress from tracking calorie intake alone can induce a stress response and increase the secretion of cortisol [160].

Research suggests that 24-hour urinary and/or plasma cortisol have been positively associated with central obesity [161, 162]. Cortisol levels are believed to peak ~20–30 minutes after waking [159]. The degree of the cortisol response to waking may be positively related to chronic perceived stress [159, 163]. Wallerius et al. [163] reported that in 53-year-old men, morning cortisol values were positively associated with body mass index, waist to hip ratio (WHR), and abdominal sagittal diameter. Similarly, another study conducted by Steptoe and colleagues [159] demonstrated that in 47 to 59 year-old men there was a significant association between waist to hip ratios and elevated cortisol response to waking (i.e., 30 minutes after waking). Interestingly, this association was not observed in women [159]. A more recent study by Larsson et al. [164] utilizing a large sample of participants (1671 men and women between 30–75 years old) measured both morning and delta cortisol (morning minus evening cortisol). It was demonstrated that salivary cortisol levels were generally higher in women as compared to men [164]. This finding is contrary to other studies that have reported that men were found to have higher cortisol levels than women [165, 166]. Furthermore, Larsson and cohort [164] reported that in women abdominal obesity was significantly associated with low morning and delta cortisol, but this was not observed in men [164]. It was hypothesized by the authors that an inverse relationship between morning cortisol and WHR in women may be due to enhanced clearance rate of cortisol in visceral fat secondary to the high density of glucocorticoid receptors [164]. Interestingly, a study by Ljung et al. [167] reported a similar inverse relationship between morning cortisol and WHR in men as well. It should be noted that some of the equivocal findings in cortisol research may, in part, be due to the differences in number of subjects in the studies, the time of day cortisol is measured, and whether cortisol was measured in the plasma, urine, or saliva [164]. Thus, it is difficult to make definitive statements regarding the differences of cortisol levels between sexes; however, it does appear that cortisol plays a role in development of central adiposity.

Knowing that perceived stress may increase cortisol levels, strategies to reduce psychological stress should be considered as a means to combat weight gain. Although regular exercise and a healthy diet are effective ways to mitigate psychological stress, the focus of this section is to describe research that has demonstrated alternative ways (i.e., nonexercise or nutrition interventions) to decrease stress and/or cortisol. Mindfulness-Based Stress Reduction (MBSR), developed at the University of Massachusetts about 30 years ago, is a program that attempts to teach people to cope more effectively with stressors [168]. The MBSR model incorporates the practice of mindfulness, meditation in a group setting, group dialogue, daily home practice, and providing theoretical material related to stress management and the mind-body connection [168]. Several studies have reported that participating in the MBSR program appears to positively affect the stress response and the HPA axis [168]. Specifically, MBSR reduced cortisol levels in certain diseased states such as cancer patients [169, 170]. However, other studies that utilized females with heart disease [171] and HIV-infected males and females [172] did not find a significant difference in cortisol levels with MSBR when compared to a control group. Although the aforementioned studies were in diseased populations and did not specifically look at weight reduction, it could be theorized that if cortisol levels could be reduced via MBSR or another similar modality, then decreases in weight may possibly result. However, more research is needed before definitive recommendations can be made. 

Ghrelin may also be affected by psychological stress, and, like cortisol, it may influence eating [157]. To date, however, less has been published regarding the effects of perceived stress on ghrelin in humans. Plasma ghrelin levels have been shown to increase approximately twofold immediately prior to a meal and decrease within an hour after the cessation of eating [135]. Similarly, in other studies, ghrelin tends to increase linearly with hunger scores [173]. Interestingly, ghrelin appears to be inversely related to BMI [174, 175]. To test the effect of stress on ghrelin, Raspopow [157] utilized the Trier Social Stress Test (TSST), which includes public speaking and an arithmetic problem in front of a panel of judges and administered it to college-aged women. The women were categorized as “emotional” or “nonemotional” eaters via a questionnaire. Blood was taken throughout the TSST. The investigators reported that regardless of eating status (i.e., emotional versus nonemotional), ghrelin levels increased moderately in response to stress [157]. However, when the participants were given food, ghrelin levels decreased significantly in the nonemotional eaters, but remained stable in the emotional eaters [157]. The authors concluded that the lack of decline of ghrelin among emotional eaters may provide a continued signal to prolong eating [157]. One could hypothesize that if this occurred chronically, it could lead to weight gain. 

Rouach et al. [174] examined the effects of psychologically induced stress on plasma ghrelin levels in patients with binge-eating disorder (BED) and in healthy subjects of normal or increased body mass index (BMI). As in the previous study by Raspopow [157], all participants were subjected to the TSST. In addition to other variables, plasma ghrelin levels were measured throughout the test. Equally important, subjects were requested to rate their urge to eat uncontrollably, subjective feelings of stress, and desire to eat sweets via a visual analog scale both prior to and after the TSST [174]. The investigators reported that after correcting for gender, age, and BMI there were no differences in ghrelin levels throughout the test among the groups or over time in each individual group [174]. However, a significant difference in ghrelin was observed after the three groups were reanalyzed according to their cortisol response to stress [174]. Specifically, ghrelin levels increased in cortisol responders, but either no change or a decrease in ghrelin levels was observed in cortisol nonresponders [174]. The authors ultimately concluded that ghrelin was independent of BMI and that psychological stress may indeed increase ghrelin levels, but it does not appear to influence the drive to eat uncontrollably or eat sweets after a stressful event [174]. 

In conclusion, due to the limited number of human studies, it is difficult to make conclusive statements regarding the effects of ghrelin on appetite and weight management. It does appear from scant evidence that perceived stress may increase ghrelin, but it is unclear whether that would translate into eating more food following a stressful event. Given exogenously via an infusion, it has been reported to increase appetite and subsequent feedings. At this point in time, however, it is not fully known whether or not stress-reducing techniques, such as the aforementioned MBSR, can significantly alter ghrelin levels, nor is it known whether chronic perturbations in ghrelin levels can appreciably affect long-term weight maintenance. More research is needed in these areas to draw concrete conclusions.

## 7. Conclusion

Although changes in the lifestyle factors discussed in this paper may result in weight control through hormone regulation and non-purposeful calorie restriction, the ultimate goal of any weight loss strategy is to expend more calories than one ingests. As is well-known, this may be accomplished by increasing exercise, by decreasing caloric intake, or by both simultaneously. More than likely negative caloric balance will have to be reached through some sort of dietary restriction considering that 30 minutes of running for a 180 pound individual results in an expenditure of only about 440 calories [176] which is just about equivalent to the calories contained in only 6 ounces of 75% fat ground beef. 

The need for novel approaches to control rates of obesity is warranted. Our paper suggests that various lifestyle factors can help attenuate the hormonal responses normally associated with appetite control and regulation. Specifically, eating more frequent and smaller meals comprised of moderate protein levels and lower fat, obtaining normal sleep of 8 hours a day, and controlling stressors and levels of psychological stress could more readily control the levels of “appetite hormones.” Additionally, it is recommended that exercise, both endurance and resistance training, should be incorporated into any lifestyle or behavior enhancement program. In closing, development of a weight loss program requires an integrative approach of many professionals including physicians, psychologists, nutritionists, and exercise physiologists who can as a team propose an optimal personalized strategy taking into account all aspects of obesity.
